# Supplementary material for: Parental bonding in retrospect and adult attachment style: A comparative study between Spanish, Italian and Japanese cultures
Source: PLoS One. 2022 Dec 1;17(12):e0278185. doi: 10.1371/journal.pone.0278185 (PMC9714759; doi:10.1371/journal.pone.0278185)
Supplement: Attachment — Submitted filename: Spanish ECR_PLOS_Response 1 - 3.0.docx [file pone.0278185.s001.docx]

Dear Reviewer, thank you for your comments. Please see our responses to your comments below.

Reviewer #1: The current manuscript makes a good contribution to the adult attachment framework. The study investigates the relations of parental bonding and adult attachment style in three different samples from 3 countries. The previous findings of the link between perceived parental bonding and attachment style have been conflicting. The current study attempts to clarify the relationships. Although the current study presents meaningful empirical cross-cultural data, I find that the information presented in the methodology, results, and discussion sections should be revised to improve cross-cultural aspects of the study before accepting for publication. Thus, my decision is to revise and resubmit. My specific feedbacks are as follows.

Abstract. I find the final sentence in this section “However, it does not exclude activation of original Internal Working Models to contexts in which the individual interprets a danger to the relationship” does not best represent many good arguments the authors have in the discussion section. The replacement of this sentence would improve the section.

Thank you for your comment. We have removed the sentence and replaced it with the following one: “These results suggest that different cultural models influence adult attachment representations differently, in terms of the weight placed on attachment-related avoidance, attachment-related anxiety, care, and overprotection in infant and adult relationships.”

Introduction. This section is well-written overall. The review of the previous findings is extensive. The rationale of the study is sound.

Thank you for your comment.

Method and results. These sections need some clarification to strengthen the cross-cultural research approach. The description of measurements used in the study needs additional information about the examination of measurement invariance to ensure the measures used are comparable across cultures. Please see Lacko et al. (2022) https://doi.org/10.1177/10693971211068971 for guideline on statistical analyses to reduce biases in cross-cultural research. The results section can be improved by adding some analyses based on this guideline as well.

Thank you for your comment. We have elaborated on the psychometric properties and factorial structures of the measures used in this study as follows:

“The PBI-S has been found to demonstrate similar psychometric properties as the PBI and was found to have a two-factor structure, although a three-factor structure appeared to show greater predictive power in relation to affective disorders [121]. The PBI-J used in [123] was demonstrated to show the same two-factor loading in [59] although there have been inconsistent findings of the factorial structure in different Japanese samples [124]. The PBI-I also demonstrated high internal consistency and a two-factor solution was also found [122].”

“The ECR-S showed the intended two-factor structure in the study conducted by Alonso-Arbiol et al. [115]. There was also evidence indicating high internal consistency, test-retest reliability and criterion and construct validity [115]. The ECR-I was also found to demonstrate the two-factor structure along with adequate internal consistency and test-retest reliability [116]. Finally, the ECR-J also demonstrated the two-factor structure and results indicated adequate internal consistency and construct validity [117].”

We have also included the analysis of measurement invariance as follows:

“First, we present the descriptive statistics, Cronbach's alpha, Kolmogorov-Smirnov normality test, and Kruskal Wallis test with Bonferroni Post-Hoc tests for ECR and PBI factors and classifications in the Spanish, Italian, and Japanese groups. To ensure ECR and PBI factors measures are comparable, we ran a multigroup factorial analysis (CFA) to test the measurement invariance and reduce possible biases in cross-cultural research [127]. Four models are explored, establishing a progressive series of restrictions on model parameters to assess the invariance between groups [128, 129]. To assess the differences between the models, we use the variations of the CFI indices (ΔCFI) and RMSEA (ΔRMSEA). The strong invariance is assumed when ΔCFI ≤ 0.01 and ΔRMSEA are ≤ 0.015 [130], while partial invariance is assumed when ΔCFI ≤ 0.01 or ΔRMSEA is ≤ 0.015 [131].”

“To explore the measurement invariance of ECR and PBI factors in the three groups, a multigroup factorial analysis (CFA) was run. The ΔCFI scores (invariance of CFA models) suggest that the ECR (F1. Anxiety; F2. Avoidance) and PBI (F1. Care; F2. Overprotection) bifactorial structures do not fit well, underlying a factor model variance in the three groups (Table 4). On the other hand, the ΔRMSEA (equivalence of metric variance) shows metric invariance in the three groups, suggesting that each item contributed to the latent construct to a similar degree across the Spanish, Italian and Japanese groups (Table 4). In conclusion, the results indicate partial invariance of the ECR metrics in the three groups.”

Table 4 - Multigroup confirmatory factorial analysis (CFA).

|  | ^①^ Model | X^2^ (Df) | X^2^/DF | ^②^ CFI | ^③^ RMSEA (IC;90%) | Comparison | ^④^ ΔX^2^ | ^⑤^ ΔCFI * | ^⑥^ ΔRMSEA* |
| --- | --- | --- | --- | --- | --- | --- | --- | --- | --- |
| ECR (F1. Anxiety; F2; Avoidance) | M1 - Unconstrained (baseline) | 4038,56 (1742) | 2,27 | 0,64 | 0,065 (0,062; 0,067) |  |  |  |  |
|  | M2 - Measurement weights | 4407,88 (1850) | 2,83 | 0,59 | 0,068 (0,065;0,070) | M2 vs M1 | 369,32 (108), p < 0,01 | -0,05 | 0,003 |
|  | M3- Measurement intercepts | 5582,30 (1922) | 2,90 | 0,42 | 0,079 (0,077;0,082) | M3 vs M2 | 1174,42 (72), p < 0,01 | -0,17 | 0,011 |
|  | M4 - Measurement residuals | 6079,85 (1988) | 3,04 | 0,36 | 0,082 (0,080; 0,085) | M4 vs M3 | 497,55 (66), p < 0,01 | -0,06 | 0,003 |
| PBI-M (F1. Care; F2; Overprot.) | M1 - Unconstrained (baseline) | 1844,46 (825) | 2,24 | 0,73 | 0,064 (0,060; 0,068) |  |  |  |  |
|  | M2 - Measurement weights | 1996,61 (871) | 2,92 | 0,71 | 0,065 (0,062; 0,069) | M2 vs M1 | 152,15 (46), p < 0,01 | -0,02 | 0,001 |
|  | M3- Measurement intercepts | 2397,89 (921) | 2,60 | 0,62 | 0,073 (0,069; 0,076) | M3 vs M2 | 431,28 (50), p < 0,01 | -0,09 | 0,008 |
|  | M4 - Measurement residuals | 2720,42 (975) | 2,79 | 0,55 | 0,077 (0,074; 0,080) | M4 vs M3 | 322,53 (54), p < 0,01 | -0,07 | 0,004 |
| PBI-F (F1. Care; F2; Overprot.) | M1 - Unconstrained (baseline) | 1767,81 (825) | 2,14 | 0,77 | 0,062 (0,058; 0,065) |  |  |  |  |
|  | M2 - Measurement weights | 1912,63 (871) | 2,20 | 0,75 | 0,063 (0,059; 0,067) | M2 vs M1 | 144,83 (46), p < 0,01 | -0,02 | 0,001 |
|  | M3- Measurement intercepts | 2381,30 (921) | 2,59 | 0,65 | 0,072 (0,069; 0,076) | M3 vs M2 | 468,66 (50), p < 0,01 | -0,1 | 0,009 |
|  | M4 - Measurement residuals | 2642,263 (975) | 2,71 | 0,60 | 0,075 (0,072; 0,079) | M4 vs M3 | 260,96 (54), p < 0,01 | -0,05 | 0,003 |
| Values indicating metric invariance are highlighted in grey.  ① M1: Unconstrained invariance model (baseline) with free estimation of factor loadings, intercepts, and error variance in each group. M2: Measurement weights model (metric invariance) with equal factor loadings in the groups. M3: Measurement intercepts model (scalar invariance) with equal intercepts and factor loadings in the groups. M4: Measurement residuals model (strict invariance) with equal intercepts, factor loadings, and error variance in the groups.  ② CFI - Comparative Fit Index; CFI ≥ 0.95 the model fits the sample [132].  ③ RMSEA - Root mean squared error of approximation; RMSEA ≤ 0,05 the model fits the sample [132].  ④ ΔX^2^ Invariance assumed when p> 0,05  ⑤ ΔCFI (between-group invariance of CFA models). Factor model invariance assumed when p< 0,01.  ⑥ ΔRMSEA (equivalence of metric variance). Metric invariance assumed when p< 0,015.  * Strong invariance is assumed when ΔCFI ≤ 0.01 and ΔRMSEA ≤ 0.015 [130]. Partial invariance is assumed when ΔCFI ≤ 0.01 or ΔRMSEA ≤ 0.015 [131]. | | | | | | | | | |

Discussion. The authors thoroughly discuss the findings related to the results section. However, more discussion should be made addressing the matter of cultural generalizations. The current manuscript indeed sounds stereotypical. This can be improved by adding points about potential cultural biases from the current study design and analysis. The small sample sizes collected from college students should be included as limitations. The results should be interpreted with cautions. The data can’t represent the three cultures.

Thank you for raising these points. We have added a section in the limitations addressing these points as follows: “Finally, the results in the present study should be interpreted with caution due to the relatively limited sample collected from each country. The participants were also sampled from university students in the three countries, which limits their generalisability to their respective cultures. Although the individualism-collectivism framework and the assumptions of Western cultures as individualistic and Eastern cultures as collectivistic is often applied in cross-cultural research, it is important to note that individualism-collectivism is continuous rather than a dichotomous categorisation and such cultural generalizations may not necessarily reflect each country as a whole. As demonstrated in a number of studies, countries themselves may not be culturally homogenous [138-139]. For example, there are significant regional differences between the Northern and Southern regions of Italy [140] and the sample in the current study was collected from a Northern autonomous province of Italy. Hence, future studies can also look to examine adult attachment within intranational subcultures as well.”
